# Who is Afraid of Cognitive Decline after Age 50 and Why: Reality or Illusion?

**DOI:** 10.1093/geroni/igaf122.2864

**Published:** 2025-12-31

**Authors:** Jacqui Smith, Marina Larkina

**Affiliations:** University of Michigan, Ann Arbor, Michigan, United States; University of Michigan, Ann Arbor, Michigan, United States

## Abstract

Tips about how to remain mentally sharp abound in the media alongside reports about the personal and societal challenges of dementia. Multiple questions remain about the impact of these messages. We use 2022 panel data from the Health and Retirement Study (HRS) to examine concerns about cognitive health and proposals suggesting a mismatch between actual cognitive performance and subjective status. Eligible participants (N = 4098: Mage = 69.5; Range 50-101; 60% women) responded to five items in the Psychosocial and Wellbeing questionnaire about concerns regarding age-related challenges [staying mentally sharp, remaining in own home, health-care expenses, communication, and developing Alzheimer’s Disease (AD)] together with subjective and objective assessments of cognition, health, perceptions of aging, and demographic covariates. Staying mentally sharp was the top concern (71%) followed by staying in one’s own home (56%) and AD (51%). The items were intercorrelated (rs = 0.62 to 0.42). Logistic regressions predicting concerns revealed that women (ORs =1.25), people with more chronic health diagnoses (ORs =1.37), and depression (ORs =1.84) were more likely to be concerned. Objective memory performance was not associated with concerns, but people who rated their memory as poor were more likely to be concerned (ORs=1.95). Additional analyses, revealed that people who viewed their own aging more positively were less likely to be concerned (OR = 0.67). In a longitudinal HRS subsample (N = 225), 31% who were not concerned about AD in a 2010 became concerned in 2022. Future research should investigate if changes in cognition, physical health, depression, and media use contribute to concerns.

